# Preferences of Physical Education Profiles Among Polish Adolescents

**DOI:** 10.3389/fpubh.2020.00466

**Published:** 2020-09-17

**Authors:** Cezary Kuśnierz, Barbara Zmaczyńska-Witek, Aleksandra M. Rogowska

**Affiliations:** ^1^Faculty of Physical Education and Physiotherapy, Institute of Physical Education, Opole University of Technology, Opole, Poland; ^2^Faculty of Social Sciences, Institute of Psychology, University of Opole, Opole, Poland

**Keywords:** adolescents, gender, physical education preferences, physical activity, school level

## Abstract

The purpose of this study was to examine the association of the preferred profiles of physical education (PE) classes with gender and school level among Polish adolescents. In the cross-sectional survey study, 1,340 Polish students (including 50% of girls) attending middle and high schools, aged between 13 and 19 years, participated. The participants selected one of four preferred profiles of PE classes. The majority (*n* = 845, 63%) of students participated in PE for “fun–pleasure–entertainment,” whereas only one third of students (*n* = 419, 31%) preferred “exercise–sweat–fitness” as a profile of PE classes. The preference for “fun–pleasure–entertainment” decreased about 41% for boys and 31% for high school students. A preference for “exercise–sweat–fitness” increased about 56% for men and 31% for high school students. Teachers should comply with students' preferences related to PE profiles, organizing PE classes in more fun-related forms for girls and in more exercise-related forms for boys. The proportions of these two preferred profiles of PE classes should turn across adolescence, increasing exercise-related forms and decreasing those fun-related ones.

## Introduction

Physical education (PE) underwent a deep transformation with the transition from a traditional sports skill orientation to a broader emphasis on health-related fitness and lifelong physical activity (PA) (1). Given the growing concerns over the PA levels of many young people and the possible health consequences, it is not surprising that the PE concept is not limited to “curriculum orientations” and teachers' educational goals, but it includes the view of students and the broader sociocultural context (2, 3). Previous studies have suggested that issues connected with the objectives of PE were well-established in theory (4–9). However, in rapidly transforming modern societies, the educational system is also undergoing significant changes; thus, there is a strong need to replicate previous research and monitor developments in this area. In light of the above, the present study is an attempt to focus on the preferences in PE classes among adolescents.

Most of the published data from different countries reported that PA decreases with an increase of age in youth (10, 11). For instance, the longitudinal prospective 10-year study by Kimm et al. (12) showed that between the ages 9–10 and 16–17 years, median habitual leisure time PA declined by 100% in African American girls (*n* = 1,213) and 64% in white girls (*n* = 1,166). In another longitudinal study by Telama and Yang (13), a decline in PA between the ages of 9 and 27 was shown in Finland, and a remarkable decline in PA level was observed after the age of 12. In a review study by Hallal et al. (14), it was found that inactivity rises with age and is increased in high-income countries. Caspersen et al. (15) also showed that PA of moderate to vigorous intensity declined consistently from age 12 in the United States, with one of the most dramatic declines appearing to occur between 13 and 18 years of age.

Youth PA behavior is influenced by biological, psychosocial, and environmental factors. Gender is identified among them as a significant correlate of PA. The vast majority of studies have consistently indicated that boys are more active than girls (11, 13, 14, 16, 17). Gender differences in various types of PA patterns (e.g., low, moderate, and vigorous) were also noticed. Some researchers point out that boys participate more in vigorous activities, while girls prefer moderate-to-low activities. Interestingly, with increasing age, boys prefer moderate- or low-activity patterns, while girls continue to participate in moderate-to-low activities (10).

One of the reasons for this gender difference may be that regular participation in sports and exercises is heavily influenced by observation of others who model these behaviors and by social reinforcement. The main emphasis is being put on parental role modeling, rules around sedentary and active pursuits, and parental support for PA (18), as well as the strong pressure from the media concerning female aesthetic models (19). On the other hand, it is often said that the school reproduces male culture and values, thus instilling a series of gender stereotypes among students, and that there are fewer opportunities for girls to participate in sports or regular exercise in some countries (20, 21).

Participating in organized PA at school and in the community is associated with greater physical effort and reduced sedentary time among both boys and girls (22). Unfortunately, PE that is currently taught in Polish schools increasingly fails to engage young people and, thus, fails to prepare them to become active creators and consumers of the varied forms of PA available outside of school. Recent research into the PA of youth in Poland has shown that only 74% of the students participated in all or almost all PE classes in the school year, while 11% took part in half or fewer of the total classes. The students' participation decreased as they got older, and the participation level was lower among the girls than the boys. Approximately 70% of the students were exempted from PE classes on written parental request, while 43% were excused on their own request. A doctor's certificate was obtained by 33% of the students, usually for a period shorter than 1 month, and 4.5% were exempted longer than 3 months. To sum up, for over 20–40% of the students, the number of PE classes in which they participated was significantly lower than the number planned by the legislator (23). Similar data were obtained in a nationwide survey carried out in the school years 2009–2010 and 2011–2012, in which, on average, the abstentions exceeded 15, 23, and 30% of students for specific levels of education (primary, middle, and high schools), respectively (24).

Low PA and PE classes' participation may largely be a function of a lack of motivation to exercise among inactive children and adolescents. Thus, teachers should aim, among other objectives, to boost adolescents' motives for the maintenance of participation in PA. Among the reasons driving adolescents to perform and to maintain physical or sportive activities, intrinsic motivation plays an important role. Intrinsic motivation refers to participation in an activity for inherent satisfaction and represents the most autonomous form of motivation (25, 26). It positively predicts the importance of students' engagement in PE, and this finally and positively predicts the intention of the student to continue doing PA (27–29). It is in accordance with the thesis that human behavior is undermined by the fulfillment of needs, self-actualization, and full realization of one's own potential, hence leading to the satisfaction with tasks at hand (30, 31).

Crum (32) distinguishes four profiles of PE classes: (1) fun–pleasure–entertainment; (2) exercise–sweat–fitness; (3) control–order–discipline; and (4) relevant learning concerning movement and sport. The PE profiles were originally intended to diagnose the quality of PE teacher education (PETE). In the current study, these PE profiles were used to examine students' preferences to be provided with an answer to the important question of which of the profiles is the strongest trigger of PE attendance and the main source of their motivation.

Although the conception and curriculum of PE should include an individual context from the students' perspective, little research was focused on the opinion of adolescent students about the preferences for the profiles of PE classes. This study examines the associations of the preferred profile of PE classes with gender and school level of students attending middle and high schools.

## Methods

### Participants

In this cross-sectional survey study, a sample of 1,340 adolescent students attending public schools in the Silesian region in the south of Poland participated. The study sample included 667 middle school students (50% of the total sample) and 673 high school students (50%), aged between 13 and 19 years. There were 675 girls (50%) and 665 boys (50%) among the participants. Table 1 shows the demographic characteristics of the sample.

**Table 1:** Demographic characteristics of the sample.

	**Girls**				**Boys**				**Total**	
	**Middle school**		**High school**		**Middle school**		**High school**			
	***n***	**%**	***n***	**%**	***n***	**%**	***n***	**%**	***n***	**%**
Type	334	24.92	341	25.45	333	24.85	332	24.78	1340	100.00
Grade										
First	111	8.28	109	8.13	111	8.28	111	8.28	442	32.98
Second	111	8.28	110	8.21	111	8.28	112	8.36	444	33.14
Third	112	8.36	122	9.10	111	8.28	109	8.13	454	33.88

### Measure

The survey asked a single question: “Which version of physical education presented below is closest to the one you would like to pursue at your school?” The students answered by selecting one of four preferred profiles of PE classes, developed by Crum (32): (1) PE as fun, pleasure, and entertainment; (2) PE as exercise, sweat, and fitness; (3) PE as control, order, and discipline; and (4) PE as relevant learning concerning movement and sport. The answers were coded 0 = *No choice* and 1 = *Choice*. Further, three close questions were added at the end of the survey, regarding such demographic variables as gender (female or male), type of school (middle or high), and grade (first, second, or third). The students answered each question by selecting one of the options.

### Procedure

The cross-sectional survey study was conducted between September 2010 and January 2011 in three voivodeships around the Silesia region (Poland): Lower Silesia, Upper Silesia, and Opole Voivodeship. The paper-and-pencil survey was administered during school time with the consent of the teachers. The research was approved by the Bioethics Committee of the Opole Medical Chamber (no. 151/2007) and conducted according to the principles of the Declaration of Helsinki. Prior to the study, written informed consent was obtained from parents of students below 16 years of age. The students were informed that the research is anonymous and voluntary and that they could withdraw from the examination at any time, without giving a reason. Data collection consisted initially of 1,355 surveys. However, 10 surveys were excluded from further analysis because of more than 10% of missing data, and five individuals refused participation in the survey. The response rate was 99%.

### Statistical Analysis

A series of Pearson's χ^2^ independence tests were performed separately to examine the relationship of the preferred PE profile with gender and school level. The null hypothesis is that the values included in rows and columns in the contingency table are equal. The alternative hypothesis (when *p* < 0.05) indicates an association between the variables. Further, binary logistic regression was conducted to discover predictors of the preferred PE profiles among such independent variables as gender (female and male) and school level (middle and high). In the univariate model of regression, gender, and school level were tested separately. Conversely, in the multivariate model, both gender and school levels were considered simultaneously as predictors. The Wald test was used to test the null hypothesis that a set of parameters is equal to zero. All statistical analyses were performed using STATISTICA 13.1 software.

## Results

**Figure 1:**
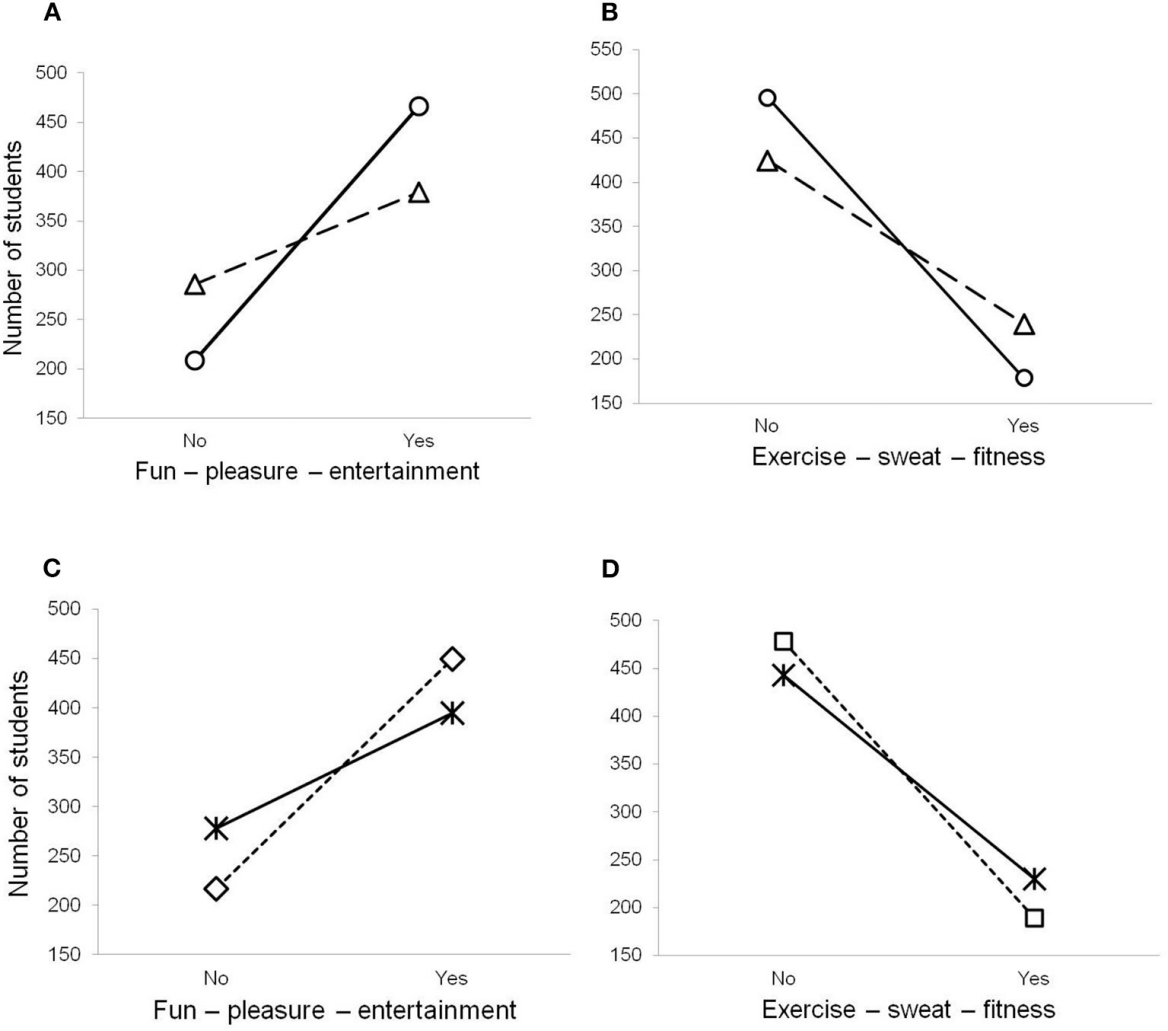
Interaction plots **(A)** between the first profile of PE classes (fun–pleasure–entertainment) and gender (female marked as a *circle* and male marked as a *triangle*); **(B)** between the second profile of PE classes (exercise–sweat–fitness) and gender (female marked as a *circle* and male marked as a *triangle*); **(C)** between the first profile of PE classes (fun–pleasure–entertainment) and school level (middle marked as a *square* and high marked as a *cross*); and **(D)** between the second profile of PE classes (exercise–sweat–fitness) and school level (middle marked as a *square* and high marked as a *cross*).

Preferences of the profiles of PE classes are shown in Table 2. Among the four profiles of PE classes, the majority of students have chosen the first (*n* = 845, 63%) and second (*n* = 419, 31%). The two other profiles of PE classes were not too common among students since only 3% (*n* = 40) has selected the fourth and 2.7% (*n* = 36) preferred the third. Table 2 contains the results of Pearson's χ^2^-test for the association between gender and school level of students and their preferences for the profiles of PE classes. Preference for the first profile of PE classes prevailed among girls, whereas boys more often chose the second profile of PE classes, as is shown in Figures 1A,B, respectively. Similar tendencies were found between students of distinct levels of schools. Students of middle school more often selected the first profile of PE classes, whereas more students of high schools have chosen the second profile, as is shown in Figures 1C,D, respectively. More boys (when compared to girls) and high school students (in comparison to middle school students) have selected the third profile of PE classes. There was neither a significant association between the fourth profile of PE classes and gender nor with school level. The interaction effect is shown in Figure 1.

**Table 2:** Contingency table and results of Pearson's χ^2^-test for the association between the four profiles of physical education, gender, and school level.

**Profiles of PE classes**	**Gender**			**School level**		
	**Female**	**Male**		**Middle**	**High**	
	***n* (%)**	***n* (%)**	**χ(1)^**2**^**	***n* (%)**	***n* (%)**	**χ(1)^**2**^**
1. Fun			20.86^***^			11.07^***^
No	209 (15.6)	286 (21.3)		217 (16.2)	278 (20.7)	
Yes	466 (34.8)	379 (28.3)		450 (33.6)	395 (29.5)	
2. Exercise			14.28^***^			5.31^*^
No	496 (37.0)	425 (31.7)		478 (35.7)	443 (33.1)	
Yes	179 (13.4)	240 (17.9)		189 (14.1)	230 (17.1)	
3. Control			5.81^*^			9.08^**^
No	664 (49.5)	640 (47.8)		658 (49.1)	646 (48.2)	
Yes	11 (0.8)	25 (1.9)		9 (0.7)	27 (2.0)	
4. Learning			0.14			0.08
No	656 (49.0)	644 (48.0)		648 (48.4)	652 (48.6)	
Yes	19 (1.4)	21 (1.6)		19 (1.4)	21 (1.6)	

* *p < 0.05*,

** *p < 0.01*,

*** *p < 0.001*.

Results of the logistic regression analyses predicting preference for the profiles of PE classes among students are shown in Table 3. Preference for the first profile of PE classes may be predicted among female and middle school students, whereas preference for the second and third profiles may be predicted among male and high school students.

**Table 3:** Results of the logistic regression analysis predicting preferences for the profiles of PE classes among students (*N* = 1,340).

**Predictors of profiles of PE classes**	**Univariate model**							**Multivariate model**						
	***B***	***SE B***	***OR***	**95%** ***CI***		**Wald's**	***p***	***B***	***SE B***	***OR***	**95%** ***CI***		**Wald's**	***p***
				***LL***	***UL***	**χ^**2**^**					***LL***	***UL***	**χ^**2**^**	
**1. Fun**														
Gender (male)	−0.52	0.11	0.59	0.47	0.74	20.72	0.00001	−0.53	0.11	0.59	0.47	0.74	21.06	0.00000
School level (high)	−0.38	0.11	0.69	0.55	0.86	11.03	0.001	−0.39	0.11	0.68	0.54	0.85	11.39	0.001
**2. Exercise**														
Gender (male)	0.45	0.12	1.56	1.24	1.98	14.19	0.0002	0.45	0.12	1.57	1.24	1.98	14.34	0.0002
School level (high)	0.27	0.12	1.31	1.04	1.66	5.31	0.02	0.28	0.12	1.32	1.05	1.67	5.45	0.02
**3. Control**														
Gender (male)	0.86	0.37	2.36	1.15	4.83	5.49	0.02	0.87	0.37	2.39	1.16	4.90	5.61	0.02
School level (high)	1.12	0.37	3.06	1.42	6.55	8.25	0.004	1.13	0.39	3.08	1.44	6.62	8.36	0.004
**4. Learning**														
Gender (male)	0.12	0.32	1.13	0.60	2.11	0.14	0.71	0.12	0.32	1.13	0.60	2.11	0.14	0.71
School level (high)	0.09	0.32	1.10	0.58	2.08	0.08	0.77	0.09	0.32	1.10	0.58	2.08	0.09	0.77

*LL, lower limit; UL, upper limit*.

## Discussion

The findings indicate that students' preferences for the profiles of PE classes are mainly related to the first (63%) and second (31%) profiles. It means that the basic, desirable forms of the activities during PE lessons are fun, leisure, entertainment, and intention to develop their physical fitness and locomotor skills. This is in line with previous research showing that positive emotions (happiness and joy) are significantly more highly evaluated than other emotions (negative and ambiguous) throughout PE sessions and that intrinsically motivated people do PA for pleasure, fun, or other self-determined reasons (25, 26, 33).

The results suggested that the preferable profile for girls and younger students from middle school was that of fun, pleasure, and entertainment, while boys and older adolescents from high school were predominantly in favor of exercise, sweat, and fitness. Previous research on gender differences in the context of PE activity support these results, as boys' more intensive PA and preferences toward vigorous activities contrasted with girls' predilection for moderate-to-low activities and lower level of activity in general (10, 11, 13, 14, 16, 17). The data also showed that the preferable profile of PE classes changes with age from the first to the second profile. Adolescence is a transitional developmental period between childhood and adulthood that is characterized by more biological, psychological, and social role changes than any other stage of life except infancy (34–36). Given the magnitude of such changes, it is not surprising that there are also significant changes in the preferred forms of physical activity and the motives and values underlying engagement in PE classes. Given the magnitude of such changes (37), it is not surprising that there are also significant changes in the preferred forms of physical activity and the motives and values underlying engagement in PE classes.

Logistic regression analysis showed that the preference for the first profile of PE classes was predicted by female gender and middle school level, whereas prediction of the occurrence of the second and third profiles concerned male gender and high school students. In other words, girls and younger students most frequently tended to choose fun, pleasure, and entertainment, whereas the group of boys and older youth preferred exercise, sweat, and fitness, as well as control, order, and discipline. A nationwide survey carried out in Polish schools showed that 14% of students expressed lack of willingness to participate in PE lessons. Consequently, from primary school to higher education, the proportion of the reluctance to attendance tended to increase: from 6% between the fourth and sixth grades of primary school, through 14% in middle school level, to 19% in high school. As one of the main reasons for the avoidance of PE classes, respondents mentioned an unattractive method of conducting classes by the teachers (31% of the total number of students) (24).

### Limitation of the Study

Despite the importance of the results of the present study, the findings must be viewed within the context of its limitations and interpreted with caution. One important limitation is the cross-sectional nature of the study, which does not allow any causal relationship of the preferred profiles of PE classes with gender and school level among adolescents. Further longitudinal research is needed to replicate the present findings. The research was performed almost a decade ago. Likely, the current and future replication of the study could have different results. The changes in PE preferences and culture may be related to generational changes. Moreover, there are other correlatives such as biological, psychosocial, and environmental factors that can affect students' preferences. Therefore, future studies need to investigate more factors related to the PE preferences. Finally, given that this research is based only on students' self-report survey, future research could combine self-report measured with a more objective qualitative method (e.g., observation).

### Conclusions

Particular Polish schools and teachers have some autonomy in developing the curriculum of physical education. Teachers' preference for selected sports disciplines may significantly affect the content of school PE. It seems necessary to also take into account students' preferences in PE practice. Students expect that PE classes will be fun and focused on recreation and entertainment. This choice dominates in both middle and high schools. Students expect physical education classes to be implemented as fun, recreation, and entertainment. This choice dominates in both types of schools. The PE curriculum close to the students' expectations may have a positive impact on the attitude toward PE participation by increasing the perceived attractiveness of PE and in engaging in physical activity and triggering greater creativity.

It appears that better understanding of students' preferences is one of the means to decrease the level of reluctance in attendance in PE classes as well as to boost the effectiveness of realizing the most important PE objectives. Organizing PE classes in more fun-related forms for girls and in more exercise-related forms for boys and using the knowledge about the changing proportions of two preferred profiles of PE classes over age in practice by increasing the exercise-related forms and decreasing the fun-related ones could provide useful guidance for teachers.

## Data Availability Statement

The datasets generated for this study are available on request to the corresponding author.

## Ethics Statement

The studies involving human participants were reviewed and approved by Bioethical Commission of the Opole Medical Chamber, 45-054 Opole, Poland. Written informed consent to participate in this study was provided by the participants' legal guardian/next of kin. Written informed consent from the participants of this study over the age of 16 was not required according to national legislation and institutional requirements.

## Author Contributions

CK, BZ-W, and AR contributed to the study conception and design, data collection and interpretation, and manuscript preparation and review. AR contributed to the statistical analysis and visualizing and writing the results. All authors contributed to the article and approved the submitted version.

## Conflict of Interest

The authors declare that the research was conducted in the absence of any commercial or financial relationships that could be construed as a potential conflict of interest.
